# A clinical trial of ropivacaine in arthocentesis for TMD

**DOI:** 10.1186/s12903-024-04606-x

**Published:** 2024-10-29

**Authors:** Laifeng Huang, Zeliu Huang, Shiyun Bi, Huaming Mai

**Affiliations:** 1 Department of Oral and Maxillofacial Surgery, College &Hospital of Stomatology, Guangxi Medical University, Nanning, Guangxi 530021 China; 2 Guangxi Key Laboratory of Oral and Maxillofacial Rehabilitation and Reconstruction, Nanning, Guangxi 530021 China; 3 Department of Stomatology, Guigang City People’s Hospital, Guigang, 537000 China; 4 Guangxi Clinical Research Center for Craniofacial Deformity, Nanning, 530021 China

**Keywords:** Temporomandibular joint disorder, Arthrocentesis, Ropivacaine, Lidocaine

## Abstract

**Introduction:**

This study aims to compare the efficacy of ropivacaine and lidocaine in the treatment of temporomandibular joint (TMJ) disorders, with the goal of exploring a more effective treatment for TMJ disorders.

**Methods:**

Patients with Wilkes stage III and IV unilateral TMJ disorders were enrolled in the study. 0.5% ropivacaine was used for local anesthesia in group A, 2% lidocaine was used in group B. Sodium hyaluronate was injected after supra-articular lavage in both groups. The patients’ general conditions, pain scores, and maximum opening before and after treatment were collected, the time of onset and maintenance of anesthesia, and the levels of inflammatory factors IL-1β and IL-6 in the joint lavage fluid were detected.

**Results:**

Study showed that the onset of anesthesia was faster and longer maintained in group A. The decrease in IL-1β was more pronounced in group A (16.08 ± 3.10) than in group B (18.03 ± 2.84), *p* < 0.05. At 2 months after treatment, the joint clicking rate was higher in group A (75%) compared to group B (35%), *p* < 0.05. At 3 months after treatment, the joint clicking rate was higher in group A (76.69%) compared to group B (40%) and the maximum mouth opening was greater in group A (45.00 ± 2.38) compared to group B (41.73 ± 4.18), *p* < 0.05. There were no statistically significant differences in VAS score and lateral excursion in group A compared with group B at 2 months and 3 months after treatment.

**Conclusions:**

Compared with lidocaine, the application of ropivacaine combined with sodium hyaluronate supra-articular lavage for the treatment of temporomandibular joint disorder is more clinically effective.

**Clinical trials Registration Number:**

ChiCTR2300075241 (30/08/2023).

**Supplementary Information:**

The online version contains supplementary material available at 10.1186/s12903-024-04606-x.

## Introduction

Temporomandibular joint disorder (TMD) is a disease affecting the bony structures and associated muscles of the temporomandibular joints. It is characterized by symptoms such as clicking of joint, muscles pain during chewing, and restricted mouth opening. [1–3] Intra-articular disorders, synovitis, and osteoarthritis are among the primary manifestations of TMD, which exhibits a prevalence ranging from 8 to 60%. Furthermore, TMD is more frequently observed in women than in men, with an average age of onset between 30 and 50 year. [1, 4, 5]

The pathogenesis of temporomandibular joint (TMJ) disorders remains unclear. Previous studies have indicated that patients with TMJ disorders exhibit the expression of interleukins IL-1β, IL-6, and tumor necrosis factor-α (TNF-α), whereas these cytokines are not detected in healthy individuals. [6, 7] Additionally, IL-1β, IL-6, and TNF-α have been found to promote the release of proteolytic and degradative enzymes and stimulate the expression of inflammatory mediators, ultimately leading to inflammation and degradation of TMJ cartilage. [8–10] Notably, IL-1β has been identified as a crucial inflammatory factor in the onset and progression of TMJ osteoarthritis. [10, 11] Based on these findings, it can be inferred that both IL-1β and IL-6 cytokines may significantly contribute to the pathogenesis of TMJ disorders.

Currently, the treatment of TMJ disorders primarily relies on conservative approaches including physical therapy, hot compresses, occlusal orthoses, and medications, such as intra-articular injections. [12] In cases where conservative treatments prove ineffective and significantly impact patients’ quality of life, surgical interventions are employed. Among these, arthrocentesis is considered a minimally invasive surgical technique for managing intra-articular TMJ disorders. It is known for its ease of performance, cost-effectiveness, and widespread clinical utilization. Arthrocentesis involves the injection of local anesthetics into the surrounding tissues of the joint area, followed by irrigation of the upper articular space to eliminate inflammatory substances from the joint cavity, resolve the “anchoring disk phenomenon,” and improve joint movement. [13–17]

Nowadays, lidocaine is commonly used as a local anesthetic agent for clinical TMJ irrigation. However, it has been noted that lidocaine exhibits insufficient strength of anesthetic effect, short duration, and significant postoperative pain. [18, 19] On the other hand, ropivacaine, a frequently used agent for oral nerve block anesthesia, offers advantages such as rapid onset of anesthesia, prolonged duration, and minimal side effects. [19] Although many studies have shown that ropivacaine can be used for intra-articular injections, there are currently no studies investigating the application of ropivacaine in human temporomandibular joint lavage. [20–22] Consequently, the objective of this study is to compare the efficacy of lidocaine and ropivacaine in TMJ disorders lavage, as well as to assess the effectiveness of ropivacaine treatment by evaluating changes in inflammatory factors IL-1β and IL-6 before and after the use of lidocaine and ropivacaine lavage. The ultimate goal is to provide a more effective treatment option for patients with TMJ disorders.

## Materials and methods

### Patients

This study recruited 49 patients diagnosed with unilateral intra-articular TMJ disorders in Wilkes staging stages III and IV. These patients underwent joint lavage performed by the same surgeon at the Department of Oral and Maxillofacial Surgery of the Affiliated Stomatological Hospital of Guangxi Medical University between November 2022 and March 2023. Regrettably, nine of these patients were lost to follow-up. All patients willingly agreed to participate in the study and provided their informed consent, which was conducted in accordance with the Declaration of Helsinki Medical Protocols and Ethics and approved by the Medical Ethics Committee of the Affiliated Stomatological Hospital of Guangxi Medical University. (2,022,076). In addition, this study passed the Chinese clinical trial registration. (Chinese Clinical Trials Registry: ChiCTR2300075241).

### Inclusion criteria

The study included patients who were at least 14 years old and met the following criteria: presence of pain, history of clicking of joint and/or joint strangulation, or symptoms of clicking of joint followed by sudden cessation and limited mouth opening. Additionally, patients with unilateral intra-articular temporomandibular joint (TMJ) disorders in Wilkes stages III and IV, as diagnosed by CBCT or MR imaging, were included.

### Exclusion criteria

Patients with a history of previous treatment for temporomandibular joint disorders, patients with rheumatoid arthritis, hematologic disorders, or other severe systemic pathologies, and patients who had allergies to local anesthetics, as well as pregnant and breastfeeding women.

### Randomization and masking

A researcher executed a simple random program based on a computer-generated table of random numbers. Participants were randomly allocated into two groups, with 20 patients in each group (*n* = 20). Group A participants received 2 ml of 0.5% ropivacaine, while Group B participants received 2 ml of 2% lidocaine. Patients were assigned to the group based on a random number table provided by the researcher for selection of random numbers and then assigned to the group based on the corresponding random numbers. Treatment allocation was concealed from the patients and surgeons.

### Variables and assessments

All patients underwent pre- and post-operative evaluations for various parameters, including heart rate and blood pressure, both before and after irrigation. Popping: The occurrence of clicking of joint during the movement of opening and closing the mouth, indicated as either “yes” or “no”. Maximum mouth opening: The vertical distance, in millimeters, between the upper and lower dentition and incisors when the patient actively opens their mouth. Lateral movement: The horizontal distance, in millimeters, between the upper and lower jaws when the mandible moves maximally towards the unaffected side. Pre- and post-operative pain levels were evaluated using the visual analog scale (VAS) for pain. Joint pain was measured using a 10-cm VAS scale, where 0 cm indicated a pain-free TMJ in rest, motion, and clinical examination, while 10 cm represented the highest level of pain experienced in any of these states. The range of 0–10 points represents varying degrees of pain: tolerable pain (1–3 points), pain affecting sleep and eating (4–6 points), and excruciating pain severely impacting daily life (7–10 points). Anesthesia effect: ①Onset time: The duration between the insertion of the injection needle into the skin and its entry into the joint cavity without causing pain, measured in seconds. ② Maintenance time: The period starting from the completion of joint irrigation until the anesthesia subsides and numbness is relieved (or pain returned).

### Clinical efficacy evaluation methods and indicators

The clinical effects of the two groups of patients were evaluated according to the following criteria at the follow-up examination after each treatment:

1. Cure: Maximum opening extent ≥ 37 mm, lateral movement ≥ 8 mm, absence of clicking of joint or murmur during mouth opening and closing, lack of pain in the joint area during mouth opening and closing.
2. Effective: Maximum opening extent less than 37 mm but greater than 30 mm, lateral movement ≥ 5 mm, presence of clicking of joint and murmur during opening and closing, VAS ≤ 3 points.
3. Improvement: Maximum opening extent less than 30 mm, lateral movement ≥ 3 mm, VAS ≤ 4 points.
4. No Improvement: Minimal change in the clinical signs and symptoms of patients post-treatment compared to pre-treatment. (5) Effective Rate = (Cure + Significant Effect + Improvement) / (Cure + Significant Effect + Improvement + No Improvement) × 100%.

### Treatment

All patients underwent a singular needle arthrocentesis, administered under local anesthesia by a skilled oral and maxillofacial surgeon. Post disinfection of the preauricular skin with 75% ethanol, group A had 2 ml of 0.5% ropivacaine extracted using a 7-gauge needle and syringe; conversely, group B had 2 ml of 2% lidocaine extracted in the same manner. The adept surgeon positioned the needle about 1 cm in front of the tragus for entry. A similar volume of either 0.5% ropivacaine or 2% lidocaine (0.5 ml) was subcutaneously administered to anesthetize the auriculotemporal nerve locally, thereby reducing the pain caused by the puncture for the patient. Following this, the needle and syringe were inserted into the superior articular cavity. The residual 1.5 ml of either 0.5% ropivacaine or 2% lidocaine was cycled ten times via the push-pull action of the syringe plunger to flush the superior articular cavity, thereby eliminating the pain caused by the flushing. This process culminated in the collection of 1.5-2 ml of flushing solution, which was then transferred to a sterile freezer tube. The samples were subsequently stored at -80 degrees Celsius for subsequent cytokine analysis. Subsequently, the superior articular cavity was flushed three times with 3 ml of 0.9% sodium chloride solution to remove as many inflammatory factors as possible. To prevent the anesthetic effect from being washed away by the saline, we performed a final flush with the anesthetic to achieve thorough anesthesia. Finally, 0.8 ml of sodium hyaluronate was injected to lubricate the articular cavity. Patients were re-evaluated after two weeks, with joint fluid from those requiring a second arthrocentesis collected identically prior to the subsequent procedure. In this research, excluding the patients lost to follow-up, all participants experienced two distinct single-needle joint punctures. Their joint fluid was collected prior to both the initial and subsequent puncture, with the latter conducted two weeks following the initial procedure.

### Cytokine analysis

The concentrations of IL-6 and IL-1β were determined using an enzyme-linked immunosorbent assay kit (ELISA) from Shanghai Yuanquan Biotechnology Center in China. The minimum measurement threshold for both cytokines was less than 0.1 pg/ml. The joint fluids were diluted with a modified standard solution and then analyzed in a test to measure the level of each cytokine. The evaluation of the joint fluid was performed based on the standard curve derived from the modified standard solution.

### Statistics

The recorded experimental data were analyzed for statistical significance using SPSS 26.0 software. Staging, gender, and popping were considered as count data and were described using the number of cases and percentages [N%]. Staging was treated as a rank variable and analyzed using the Wilcoxon rank sum test. Gender and popping were tested using the χ 2 test. The pre- and post-injection heart rate, blood pressure, anesthesia onset time, and maintenance time squares were analyzed using the paired t-test. The measurement information was presented as `x ± s. The t-test was employed for intergroup comparisons, while repeated measures ANOVA was utilized for analyzing two-way repeated measures between groups. The comparison of effective rates between the two groups was conducted using the ridit rank-sum test. A significance level of *P* < 0.05 was considered indicative of a statistically significant difference.

## Results

For this study, we recruited a total of 49 individuals who were subsequently randomized into two groups: Group A (ropivacaine group, *n* = 20) and Group B (lidocaine group, *n* = 20). Nine participants were excluded from the final analysis due to their non-participation in any follow-up evaluations. The reasons for exclusion were out-of-town schooling or work commitments (seven participants), and complete recovery of symptoms post-initial treatment (two participants). Consequently, our final sample comprised 40 participants who successfully completed the 2-month follow-up: 20 from Group A and 20 from Group B. Additionally, 28 participants underwent a 3-month follow-up, with 13 from Group A and 15 from Group B. We evaluated patients based on TMD stage, heart rate, blood pressure, pain intensity, maximal mouth opening, lateral movement, clicking of joint, anesthesia effect, changes in IL-1β, IL-6 before and after treatment, as well as treatment efficacy at baseline and at 2 and 3 months post-treatment.

### Patient characteristics

The mean age of the sample population in this study was 26.12 ± 12.9 years, with a gender distribution of 12.5% males and 87.5% females. Specifically, Group A consisted of 17 females (85.00%) and 3 males (15.00%), while Group B comprised 18 females (90.00%) and 2 males (10.00%). Among the participants, 21 (52.50%) were classified as Wilkes stage III (9 from Group A and 12 from Group B), and 19 (47.50%) were categorized as Wilkes stage IV (11 from Group A and 8 from Group B). We compared heart rate and blood pressure before and after injection between the groups. In Group A, the average heart rate was 74.85 ± 9.98 beats/min before injection and 74.80 ± 8.70 beats/min post-injection. Conversely, in Group B, it was 77.50 ± 12.67 beats/min pre-injection and 72.95 ± 10.20 beats/min post-injection. The mean blood pressure in Group A was recorded as 108.80 ± 14.34/69.70 ± 10.19 mmHg prior to injection and 109.85 ± 14.91/72.15 ± 12.35 mmHg after. In Group B, these values were 109.32 ± 12.33/66.20 ± 8.84 mmHg before injection and 108.50 ± 12.37/69.20 ± 9.54 mmHg post-injection. No significant differences were observed between Group A and B in terms of age, gender, TMD Wilkes stage, heart rate, and blood pressure before and after injection (Table 1).

**Table 1:** Baseline demographic and clinical characteristics of participants

Variables	Group	Number	Mean ± Standard Deviation	*P*-value
Age(yr)	A	20	25.5 ± 11.9	*P* = 0.764
	B	20	26.8 ± 14.0	
Sex M/F	A	3/17		*P* = 0.633
	B	2/18		
Wilkes stage, n (%) III IV	A	9(45%)		*P* = 0.348
	B	12(60%)		
	A	11(55%)		
	B	8(40%)		
Pre-treatment heart rate	A	20	74.85 ± 9.98	*P* = 0.467
	B	20	77.50 ± 12.67	
Post-treatment heart rate	A	20	74.80 ± 8.70	*P* = 0.541
	B	20	72.95 ± 10.20	
Pre-treatment systolic blood pressure	A	20	108.80 ± 14.34	*P* = 0.905
	B	20	109.32 ± 12.33	
Post-treatment systolic blood pressure	A	20	109.85 ± 14.91	*P* = 0.757
	B	20	108.50 ± 12.37	
Pre-treatment diastolic blood pressure	A	20	69.70 ± 10.19	*P* = 0.253
	B	20	66.20 ± 8.84	
Post-treatment diastolic blood pressure	A	20	72.15 ± 12.35	*P* = 0.403
	B	20	69.20 ± 9.54	

Abbreviation: VAS, Visual Analog Scale

### Assessment of pain levels

**Fig. 1:**
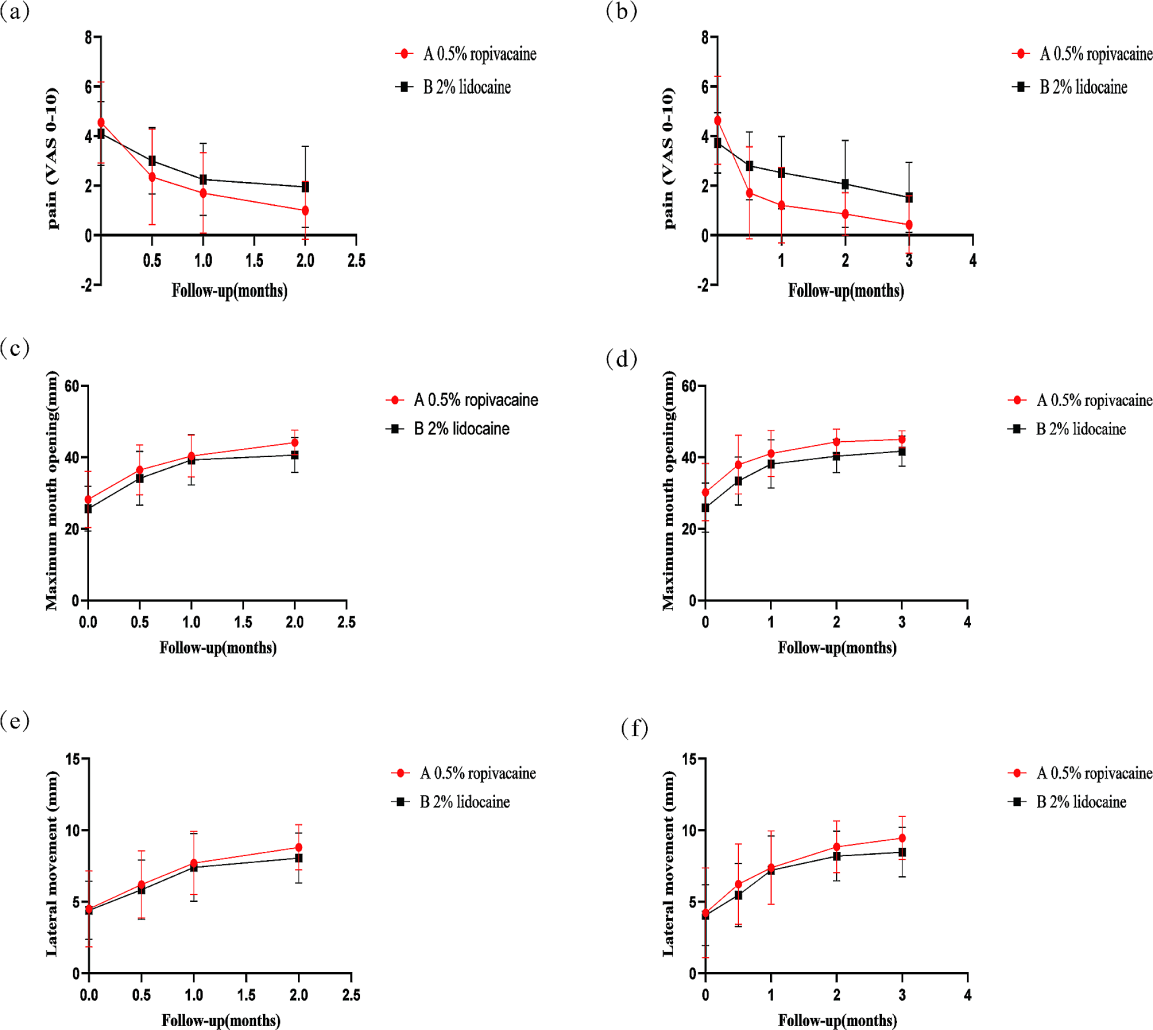
Comparative Analysis of Visual Analogue Scale (VAS) Scores, Maximum Opening, and Lateral Movement Across all Groups. A group, 0.5% ropivacaine; B group,2% lidocaine. The number of people followed up at 2 months is *n* = 40, and the number of people followed up at 3 months is *n* = 28. (**a**) Pain reports at different times during the 2-month follow-up. (**b**) Pain reports at different times during the 3-month follow-up. (**c**) Maximum mouth opening reports at different times during the 2-month follow-up. (**d**) Maximum mouth opening reports at different times during the 3-month follow-up. (**e**) Lateral movement reports at different times during the 2-month follow-up. (**f**) Lateral movement reports at different times during the 3-month follow-up.t0: Before the first injection; t1: Before the second injection; t2: After one month post-injection t3: After two months post-injection

The impact of anesthetic injections on pain reduction in both groups was examined using repeated measures analysis of variance. Pain levels were observed to decrease in both groups, prior to the first (t0) and second injections (t1), as well as one (t2), two (t3), and three months (t4) post-injection. The decline in pain levels at varying time points during the two-month (*p* < 0.001) and three-month (*p* < 0.001) follow-ups proved statistically significant. Nevertheless, when contrasting Groups A and B, no meaningful difference in pain levels was discerned during the two-month (*p* = 0.227) or three-month (*p* = 0.118) follow-ups. Indeed, our examination of different time points revealed that at t3 (two-month follow-up), the decrease in pain levels was statistically significant (*P* < 0.05) for both groups, with Group A displaying a larger reduction. The decline in pain levels at t3 (three-month follow-up) was statistically significant (*P* < 0.05) for both groups. Moreover, at t2, t3, and t4 during the three-month follow-up, the decrease in pain level remained statistically significant (*P* < 0.05) in both groups, with Group A continuing to exhibit a greater reduction (Fig. 1, Table S1, S2).

### Assessment of jaw movements

Before treatment, there was no significant difference between groups A and B in the mean value of measurements, including maximum mouth opening and lateral movement, at either 2 or 3 months of follow-up (*P* = 0.256, 0.895, 0.138, 0.871, respectively). At 2 months of follow-up, the improvement of maximum opening and lateral movement between group A and group B was not statistically significant (*p* = 0.134, 0.512, respectively), but the improvement of maximum opening and lateral movement between group A and group B was statistically significant at different time points (*p* = 0.000, 0.000, respectively), and at t3, The improvement of the maximum opening of group A and group B was statistically significant (*p* = 0.013), and the maximum opening of group A was larger than that of group B. At the follow-up of 3 months, the improvement of lateral movement between group A and group B was not statistically significant (*p* = 0.416), but the improvement of maximum opening in group A and group B was statistically significant (*p* = 0.032). A comparative summary of the results is given in Fig. 1, Table S1, S2.

### Assessment of clicking of joint

Our study revealed that prior to the initial injection (t0), before the subsequent injection (t1), and one month post-injection (t2), there was no significant incidence of clicking of joint in either group. However, two months (t3) and three months (t4) post-injection, a statistically significant increase in clicking of joint was observed in both groups (*p* < 0.05). This difference was elucidated through percentage comparison. Two months post-treatment, group A displayed a popping frequency of 75%, markedly higher than the 35% exhibited by group B; three months post-treatment, group A reported a popping frequency of 76.92%, significantly greater than the 40.00% noted in group B, with a statistical difference at *p* < 0.05 between the two groups (Table 2).

**Table 2:** Statistics of clicking changes in the first 2 and 3 months of enrolled patients

Time Two months	clicking yes/no	Group		χ^2^	*p*
		A(%)	B(%)		
t0	no	18(90.00)	19(95.00)	0.360	0.548
	yes	2(10.00)	1(5.00)		
t1	no	16(80.00)	18(90.00)	0.784	0.376
	yes	4(20.00)	2(10.00)		
t2	no	8(40.00)	12(60.00)	1.600	0.206
	yes	12(60.00)	8(40.00)		
t3	no	5(25.00)	13(65.00)	6.465	0.011*
	yes	15(75.00)	7(35.00)		
Three months	no	3(23.08)	9(60.00)	3.877	0.049*
	yes	10(76.92)	6(40.00)		

* *p* < 0.05 ** *p* < 0.01 Total number of people followed up for 2 months *n* = 40, Total number of people followed up for 3 months: *n* = 28

### Assessment of anesthesia effect

Regarding the evaluation of anesthesia effect, our study demonstrated that the onset time for anesthesia was 44.75 ± 21.24s, while the maintenance duration was 329.25 ± 47.30 min in group A. Conversely, in group B, the anesthesia onset time was recorded as 89.4 ± 36.23 s, and the maintenance duration was 175.50 ± 44.04 min. A statistically significant disparity was observed concerning the anesthesia onset time (s) between the two groups (*p* = 0.000), and a similar statistical variation was found for the anesthesia maintenance duration (min) (*P* = 0.000). These results indicate that group A experienced a shorter onset time for anesthesia and an extended maintenance duration (Table 3).

**Table 3:** Comparison of anesthesia onset time and maintenance duration across all groups

Variables	Group	number	Mean ± Standard Deviation	*P*-value
Time of onset of anesthesia(s)	A	20	44.75 ± 21.24	0.000**
	B	20	89.4 ± 36.23	
Anesthesia maintenance time(min)	A	20	329.25 ± 47.30	0.000**
	B	20	175.50 ± 44.04	

### Changes in IL-1β and IL-6 pre- and post-treatment

**Fig. 2:**
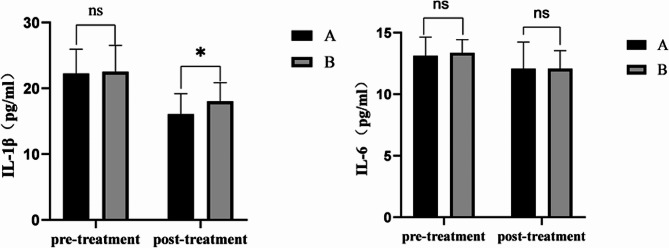
Difference of cytokines levels in the synovial fluids of the two groups. (**a**) IL-1β expression levels in synovial fluid. (**b**) IL-6 expression levels in synovial fluid. A group(*n* = 20), 0.5% ropivacaine; B group(*n* = 20),2% lidocaine

Prior to treatment, the concentrations of IL-6 (13.13 ± 1.52 pg/ml) and IL-1β (22.25 ± 3.69 pg/ml) were observed in patient samples from group A. Following treatment, the respective values were IL-6 (12.08 ± 2.16 pg/ml) and IL-1β (16.08 ± 3.10 pg/ml). In contrast, group B’s pre-treatment concentrations of IL-6 and IL-1β were 13.37 ± 1.06 pg/ml and 22.53 ± 3.99 pg/ml respectively, with post-treatment levels being IL-6 (12.08 ± 1.46 pg/ml) and IL-1β (18.03 ± 2.84 pg/ml). Prior to injection, no statistically significant difference was detected between the two groups concerning IL-6, IL-1β, and post-injection IL-6 expressions. However, a statistically significant disparity emerged post-injection in the expression of IL-1β between the two groups (*p* < 0.05), with the mean value in group A (16.08) significantly lower than that of group B (18.03) (Fig. 2, Table S3.)

### Evaluation of treatment effect

In assessing the efficacy of the treatment, a 2-month follow-up indicated an effectiveness rate of 100% for group A, compared to 65% for group B. The Ridit analysis was employed to evaluate the differences in treatment outcomes between groups A and B. There was a statistically significant difference in therapeutic effect (z = 3.478, *p* = 0.001 < 0.05), with group A exhibiting superior results to group B. By the third month, both groups recorded an effective rate of 100%, rendering the difference in treatment effect between the two groups statistically insignificant (z = 0.586, *p* = 0.558 > 0.05) (Table 4, S4, S5).

**Table 4:** Results of ridit analysis of 2-month and 3-month cure rate of enrolled patients

Group	Mean Ridit Value	95% CI	z	*p*
Two months				
A	0.659	0.532 ~ 0.785	3.478	0.001**
B	0.341	0.215 ~ 0.468		
Three months				
A	0.534	0.377 ~ 0.691	0.586	0.558
B	0.470	0.324 ~ 0.616		

*p < 0.05 ** *p* < 0.01

## Discussion

In recent years, temporomandibular joint arthrocentesis has been gradually utilized for the treatment of temporomandibular joint disorders due to its simplicity and minimally invasiveness. An increasing body of robust evidence suggests that arthrocentesis, coupled with intra-articular sodium hyaluronate injections, can effectively treat TMJ disorders and significantly enhance joint function. [23–27] Currently, the most prevalent local anesthetic used in human TMJ arthrocentesis is lidocaine. The utilization of ropivacaine is primarily limited to knee joints, with no reported applications in human TMJ arthrocentesis. Therefore, this study is the first study to evaluate the therapeutic effect of ropivacaine versus lidocaine for joint lavage in temporomandibular joint disorder. Our results showed that the clinical efficacy of ropivacaine combined with sodium hyaluronate supra-articular lavage in the treatment of temporomandibular joint disorders was more significant compared to lidocaine. This was demonstrated by the rapid onset and maintenance of anesthesia, reduced levels of the inflammatory factor IL-1β, as well as a high popping rate and maximum mouth opening.

Ropivacaine, an amide class local anesthetic, exhibits clinical efficacy similar to bupivacaine and lower cardiotoxicity. Lidocaine, another member of the amide group, has approximately half the action duration of bupivacaine. [28–30] This observation aligns with our study’s findings, which identified a significant difference in the onset and maintenance of anesthesia between patient groups injected with ropivacaine and lidocaine. The ropivacaine group experienced quicker anesthesia onset and longer maintenance periods. Existing research indicates that all these local anesthetics can be used for intra-articular injections, predominantly lidocaine, but they are chondrotoxic. However, ropivacaine’s toxicity is concentration-dependent, becoming toxic at concentrations exceeding 0.75%. [20, 31] In our investigation, we employed a ropivacaine concentration of 0.5% for supra-articular cavity irrigation, followed by saline irrigation and sodium hyaluronate injection, which reduced the ropivacaine concentration to non-toxic levels. It has also been documented that lidocaine exhibits chondrotoxicity regardless of its concentration. Yazdi et al. found that 2% lidocaine inhibited chondrocyte activity through intra-articular injection into rabbit knee joints. [32] Karpie and Chu demonstrated dose and time-dependent cytotoxic effects of lidocaine on bovine articular chondrocytes. [33] Moreover, research indicates that the combination of lidocaine and hyaluronic acid inhibits lidocaine-induced apoptosis in human chondrocytes in vitro through P53-dependent mitochondrial apoptotic pathway inhibition, thereby attenuating lidocaine-induced chondrotoxicity. [34] Though ropivacaine causes chondrotoxicity when injected into synovial joints, this toxicity is not evident until the concentration exceeds 0.75%. Grishko et al. [35]. showed that 2% lidocaine may be more toxic than 0.5% ropivacaine and 0.5% bupivacaine. Also Benjamin et al. found that the local anesthetics ropivacaine, bupivacaine, and 1% or 2% lidocaine treatment induced cellular damage in human chondrocytes in vitro. Whereas ropivacaine appears to be a local anesthetic with the lowest toxicity to human chondrocytes, this characteristic may favor its preferential use for joint injections. [36].

In a randomized, double-blind study involving 72 ASA I-II patients scheduled for elective knee arthroscopy under general anesthesia, intra-articular injection of varying doses of ropivacaine (100 mg, 150 mg, 200 mg) and bupivacaine (100 mg) was administered into the knee joint at the conclusion of the procedure. The results indicated that verbal pain scores were lower in the ropivacaine 150 mg group compared to the bupivacaine 100 mg group. Furthermore, all doses of ropivacaine showed a tendency towards reduced analgesic consumption and lower pain scores. [21] Mandal et al. deduced that intra-articular injection of ropivacaine resulted in improved postoperative pain relief, extended the duration to first analgesia, and decreased the demand for postoperative analgesia relative to fentanyl and dexmedetomidine. [22] These findings align with our study results where we observed statistically significant lower pain scores at different time points in the ropivacaine group compared to the lidocaine group. We also noted no significant difference in pain levels between the two groups during the two and three-month follow-up periods (*p* > 0.05). Nevertheless, the mean pain scores remained lower in the ropivacaine group than in the lidocaine group, a discrepancy potentially attributable to inadequate follow-up time.

It is worth noting that our study also found that the expression of IL-1β in both ropivacaine and lidocaine groups was decreased, and the expression of IL-1β in joint fluid after treatment in both groups was statistically significant (*p* < 0.05), and the expression of IL-1β in ropivacaine group was significantly lower than that in lidocaine group. This corresponds with the findings of Rabinow et al., who observed a notable reduction in pain and simultaneous decrease in cytokine IL-1β following intra-articular injection of a novel ropivacaine crystalline micromix into a peptidoglycan (PGPS)-induced swollen ankle joint using a rat model. [37] However, in our study, there was no statistical difference in IL-6 expression between ropivacaine and lidocaine groups before and after treatment (*p* > 0.05). The subsequent studies could elucidate these findings. In an experimental study comparing experimentally induced osteoarthritis with control joints in a canine model, a significant elevation of IL-6 was observed in almost all experimental animals at an early stage (3 months) compared to control. [38] This could be attributed to IL-6 expression only occurring in acute, localized, inflammatory joint processes. The patients included in our study were in stages III and IV of unilateral intra-TMJ disorders according to Wilkes staging and were not in early or acute stages, which may explain the lack of difference in the expression of IL-6 pre- and post-treatment between the two patient groups treated with ropivacaine and lidocaine. The insufficient sample collection in our study could also account for this outcome.

The current research substantiates that the intra-articular injection of sodium hyaluronate post-TMJ arthrocentesis mitigates pain and enhances maximum mouth opening. Our findings concur with this observation. It is noteworthy that at a three-month follow-up, both the ropivacaine and lidocaine patient groups exhibited a statistically significant improvement in maximum opening. However, the ropivacaine group showed superior enhancement compared to the lidocaine group. As for the assessment of clicking of joint, our study showed that the rate of clicking of joint in patients in the ropivacaine group was higher than that of lidocaine at 3 months after treatment and was statistically significant between the two groups. This is due to the fact that the temporomandibular joint disc was in irreducible anterior displacement at the time of the patient’s initial presentation, and the clicking of joint went from positive to nonpositive. After treatment the patient’s clicking of joint resumed, indicating that the TMJ disc went from being in irreducible anterior disc displacement to reducible disc displacement, which suggests to us that in TMJ arthrocentesis, the use of ropivacaine is more effective than lidocaine.

While the findings of this study furnish insightful data for refining TMJ disorder treatments, it is important to acknowledge its limitations. Initially, the small sample size could potentially limit the broad applicability of the outcomes. And because of force majeure reasons, patients could not come for follow-up on time, so this study could only compare the data for the number of patients followed up for 2 and 3 months, respectively. However, this may lead to inconsistencies in the data collected at different times.

Additionally, the brief follow-up period may not have fully captured the long-term effects of the treatment. Consequently, future research could substantiate and fine-tune the effectiveness of ropivacaine in treating TMJ disorders by enlarging the sample size, prolonging the follow-up duration, and incorporating more diverse patient attributes. Ultimately, our research might impact the selection of therapeutic strategies for TMJ disorders, leading clinicians towards a preference for ropivacaine.

## Conclusion

Both ropivacaine and lidocaine significantly ameliorate symptoms in temporomandibular joint disorder patients undergoing joint lavage. However, ropivacaine demonstrates superior efficacy in anesthetic strength, enhancing mouth opening, and pain relief. Furthermore, post-lavage IL-1β levels in the joint fluid diminish substantially with ropivacaine compared to the lidocaine group, accompanied by marked symptom improvement. This suggests that ropivacaine is more beneficial in lowering IL-1β content and mitigating joint inflammation, thereby making it a commendable clinical treatment option.

## Electronic supplementary material

Below is the link to the electronic supplementary material.

Supplementary Material 1

## Data Availability

The datasets generated and/or analyzed during the current study are not publicly available due to ethical concerns but are available from the corresponding author on reasonable request.

## Abbreviations

TMD: Temporomandibular disorder
TMJ: Temporomandibular joint
VAS: visual analog scale
