# Supplementary material for: A clinical trial of ropivacaine in arthocentesis for TMD
Source: BMC Oral Health. 2024 Oct 29;24:1311. doi: 10.1186/s12903-024-04606-x (PMC11520862; doi:10.1186/s12903-024-04606-x)
Supplement: Supplementary file 1 — Supplementary Material 1 [file 12903_2024_4606_MOESM1_ESM.doc]

**Supplemental material**


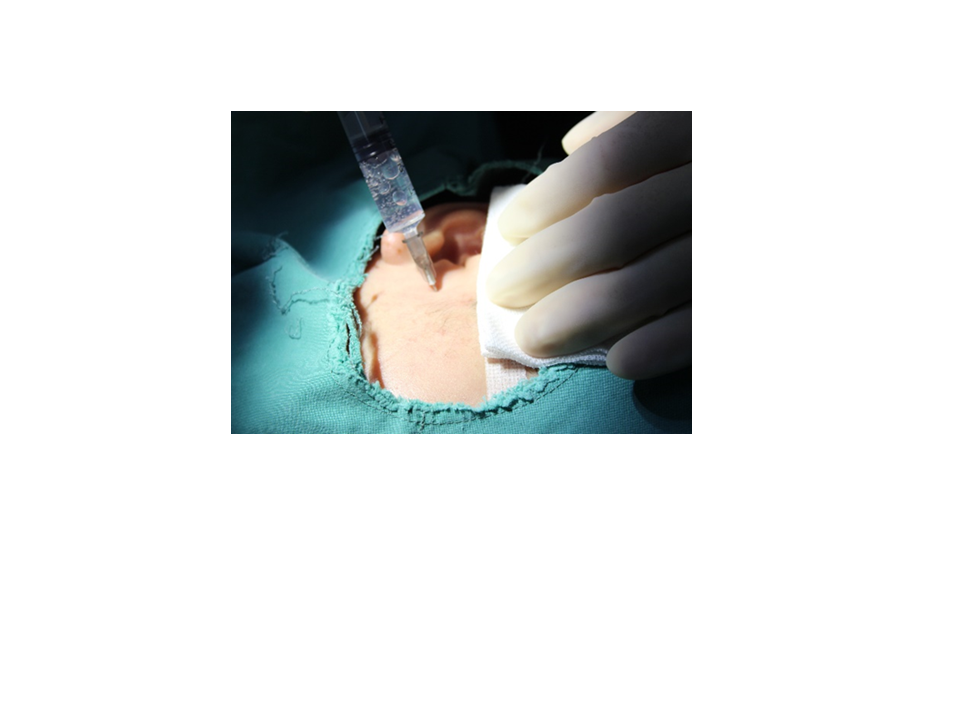


Figure S1:Tempromandibular joint arthrocentesis

Table S1: Two-Month Comparative Analysis of Visual Analogue Scale (VAS) Scores, Maximum Opening, and Lateral Movement Across all Groups

| Time | VAS(mean±SD) | | P-value | Maximum opening (mm) (mean±SD) | | P-value | Lateral movement (mm)  (mean±SD) | | P-value |
| --- | --- | --- | --- | --- | --- | --- | --- | --- | --- |
|  | A | B |  | A | B |  | A | B |  |
| t0 | 4.55±1.64 | 4.10±1.29 | 0.341 | 28.20±7.87 | 25.60±6.29 | 0.256 | 4.50±2.67 | 4.40±2.04 | 0.895 |
| t1 | 2.35±1.93 | 3.00±1.34 | 0.223 | 36.45±6.99 | 34.10±7.53 | 0.313 | 6.20±2.35 | 5.85±2.08 | 0.621 |
| t2 | 1.70±1.63 | 2.25±1.45 | 0.265 | 40.35±5.87 | 39.25±7.03 | 0.594 | 7.70±2.20 | 7.40±2.37 | 0.681 |
| t3 | 1.00±1.17 | 1.95±1.64 | 0.041 | 44.15±3.47 | 40.65±4.86 | 0.013 | 8.80±1.58 | 8.05±1.76 | 0.164 |
| Repeated measure design analysis | Group  Time  Interaction | | 0.227 | Group  Time  Interaction | | 0.134 | Group  Time  Interaction | | 0.512 |
|  |  |  | 0.000 |  |  | 0.000 |  |  | 0.000 |
|  |  |  | 0.09 |  |  | 0.668 |  |  | 0.729 |

Table S1. Two-Month Comparative Analysis of Visual Analogue Scale (VAS) Scores, Maximum Opening, and Lateral Movement Across all Groups. A group(n=20), 0.5% ropivacaine; B group(n=20),2% lidocaine. (a )Pain reports at different times.(b)Maximum mouth opening reports at different times. (c) Lateral movement reports at different times. t0: Before the first injection; t1: Before the second injection; t2: After one month post-injection t3: After two months post-injection.

Table S2: Three-Month Comparative Analysis of Visual Analogue Scale (VAS) Scores, Maximum Opening, and Lateral Movement Across all Groups

| Time | VAS(mean±SD) | | P-value | Maximum opening (mm) (mean±SD) | | P-value | Lateral movement (mm)  (mean±SD) | | P-value |
| --- | --- | --- | --- | --- | --- | --- | --- | --- | --- |
|  | A | B |  | A | B |  | A | B |  |
| t0 | 4.64±1.78 | 3.73±1.22 | 0.118 | 30.23±8.00 | 25.93±6.87 | 0.138 | 4.23±3.14 | 4.07±2.12 | 0.871 |
| t1 | 1.71±1.86 | 2.8±1.37 | 0.083 | 37.92±8.19 | 33.4±6.74 | 0.121 | 6.23±2.80 | 5.47±2.20 | 0.427 |
| t2 | 1.21±1.53 | 2.53±1.46 | 0.025 | 41.08±6.47 | 38.13±6.70 | 0.249 | 7.38±2.57 | 7.20±2.40 | 0.846 |
| t3 | 0.86±1.29 | 2.07±1.75 | 0.045 | 44.31±3.57 | 40.33±4.64 | 0.019 | 8.85±1.82 | 8.20±1.74 | 0.346 |
| t4 | 0.43±1.16 | 1.53±1.41 | 0.029 | 45.00±2.38 | 41.73±4.18 | 0.020 | 9.46±1.51 | 8.47±1.73 | 0.119 |
| Repeated measure design analysis | Group |  | 0.087 | Group |  | 0..032 | Group |  | 0.416 |
|  | Time |  | 0.000 | Time |  | 0.000 | Time |  | 0.000 |
|  | Interaction |  | 0.004 | Interaction |  | 0.902 | Interaction |  | 0.385 |

Table S2. Three-Month Comparative Analysis of Visual Analogue Scale (VAS) Scores, Maximum Opening, and Lateral Movement Across all Groups. A group(n=13), 0.5% ropivacaine; B group(n=15),2% lidocaine.(a )Pain reports at different times.(b)Maximum mouth opening reports at different times. (c) Lateral movement reports at different times. t0: Before the first injection; t1: Before the second injection; t2: After one month post-injection t3: After two months post-injection. t4: After three months post-injection.

Table S3 Statistical table of IL-6 and IL-1β changes before and after injection

| IL-6、IL-1β（pg/ml） | Group | | *t* | *p* |
| --- | --- | --- | --- | --- |
|  | A | B |  |  |
| Pre-injection IL-6 | 13.13±1.52 | 13.37±1.06 | -0.583 | 0.564 |
| Post-injection IL-6 | 12.08±2.16 | 12.08±1.46 | 0.004 | 0.997 |
| Pre-injection IL-1β | 22.25±3.69 | 22.53±3.99 | -0.227 | 0.822 |
| Post-injection IL-1β | 16.08±3.10 | 18.03±2.84 | -2.082 | 0.044* |

Table S4 Statistical table of treatment in the first 2 months of enrollment

| Group | cure | Significant effect | improvement | No improvement | Effective rate |
| --- | --- | --- | --- | --- | --- |
| A | 1 | 18 | 1 | 0 | 100% |
| B | 0 | 7 | 6 | 7 | 65% |
| overall | 1 | 25 | 7 | 7 | 82.5% |

Table S5 Statistical table of treatment status of enrolled patients at the third month

| Group | cure | Significant effect | improvement | No improvement | Effective rate |
| --- | --- | --- | --- | --- | --- |
| A | 2 | 10 | 1 | 0 | 100% |
| B | 1 | 12 | 2 | 0 | 100% |
| overall | 3 | 22 | 3 | 0 | 100% |
